# Soothing Your Heart and Feeling Connected: A New Experimental Paradigm to Study the Benefits of Self-Compassion

**DOI:** 10.1177/2167702618812438

**Published:** 2019-02-06

**Authors:** Hans Kirschner, Willem Kuyken, Kim Wright, Henrietta Roberts, Claire Brejcha, Anke Karl

**Affiliations:** 1 Mood Disorder Centre, College of Life and Environmental Sciences, University of Exeter; 2 Department of Psychiatry, University of Oxford; 3 Institute of Psychology, Otto-von-Guericke University

**Keywords:** self-compassion, self-criticism, psychophysiology, positive affiliative affect

## Abstract

Self-compassion and its cultivation in psychological interventions are associated with improved mental health and well-being. However, the underlying processes for this are not well understood. We randomly assigned 135 participants to study the effect of two short-term self-compassion exercises on self-reported-state mood and psychophysiological responses compared to three control conditions of negative (rumination), neutral, and positive (excitement) valence. Increased self-reported-state self-compassion, affiliative affect, and decreased self-criticism were found after both self-compassion exercises and the positive-excitement condition. However, a psychophysiological response pattern of reduced arousal (reduced heart rate and skin conductance) and increased parasympathetic activation (increased heart rate variability) were unique to the self-compassion conditions. This pattern is associated with effective emotion regulation in times of adversity. As predicted, rumination triggered the opposite pattern across self-report and physiological responses. Furthermore, we found partial evidence that physiological arousal reduction and parasympathetic activation precede the experience of feeling safe and connected.

Self-compassion has been defined as being kind to one’s self (Neff, 2003b) and being able to use self-reassurance and soothing in times of adversity (Gilbert, 2009; Neff, 2003b). It includes being nonjudgmental about one’s self (Gilbert, 2009; Neff, 2003b) and recognizing one’s experience as part of the human condition (Neff, 2003b). Self-criticism, on the other hand, is characterized by maladaptive emotion-regulation strategies such as being harsh and judgmental about one’s self (Gilbert, 2009; Neff, 2003b). It is associated with feeling isolated (Neff, 2003b) and being in flight or fight or social rank mode, therefore exacerbating a sense of threat in difficult times (Gilbert, 2009).

Whereas self-criticism has been associated with a number of mental health problems, such as depression and anxiety (Clark, Watson, & Mineka, 1994), there is growing evidence that self-compassion has beneficial effects on mental health and well-being (e.g., Galante, Galante, Bekkers, & Gallacher, 2014) from two lines of research. Cross-sectional, correlational studies investigating the associations between dispositional levels of self-compassion and psychological health using the Self-Compassion Scale (Neff, 2003a) revealed that higher levels of trait self-compassion are associated with higher levels of well-being (Zessin, Dickhauser, & Garbade, 2015) and quality of life (Wei, Liao, Ku, & Shaffer, 2011), health-related behaviors such as exercising (Magnus, Kowalski, & McHugh, 2010) or seeking medical treatment (Terry & Leary, 2011), and enhanced interpersonal functioning (Neff, 2003a; Neff & Beretvas, 2013). In contrast, lower levels of self-compassion were associated with mental health problems such as posttraumatic stress disorder (Thompson & Waltz, 2008) and depression (Kuyken et al., 2015). The correlational nature of these studies prevents causal conclusions of these associations.

A better understanding of the possible directionality comes from experimental and clinical studies assessing the effects of psychological interventions that directly or indirectly cultivate self-compassion and identify associated changes in psychological health. For example, kindness-based meditations drawing from Buddhists traditions, such as loving-kindness meditation (i.e., an exercise oriented toward enhancing unconditional kindness toward oneself and others), have been found to cultivate self-compassion (Galante et al., 2014; Neff & Germer, 2013) and self-acceptance (Fredrickson, Cohn, Coffey, Pek, & Finkel, 2008), increase positive (Fredrickson et al., 2008; Hofmann, Grossman, & Hinton, 2011; Klimecki, Leiberg, Lamm, & Singer, 2013; Kok et al., 2013) and decrease negative affect (Hofmann et al., 2011; Klimecki et al., 2013), increase empathy or warmth toward others (Ashar et al., 2016; Klimecki et al., 2013), and increase social connectedness (Hutcherson, Seppala, & Gross, 2008; Kok et al., 2013). In addition, mindfulness-based cognitive therapy (MBCT), an 8-week psychosocial program particularly designed for the treatment of depressive relapse (Segal, Teasdale, & Williams, 2002; Segal, Williams, & Teasdale, 2013), has been shown to increase self-compassion, which in turn predicted well-being 15 months later (Kuyken et al., 2010). MBCT uses mindfulness practices such as the body scan^1^ and breath awareness^2^ to teach mindfulness skills. Interestingly, even though it is not an explicit skill taught in MBCT, self-compassion is implicitly interwoven into the mindfulness instructions (e.g., “Whenever you notice that the mind has wandered off, bring it back with gentleness and kindness.”). This suggests that self-compassion can also be cultivated via more indirect interventions. Although it is unknown if the directness of the intervention is associated with differential processes, this may be important because direct cultivation of self-compassion could be more challenging when there is an underlying psychopathology such as depression (Gilbert, McEwan, Matos, & Rivis, 2010). Therefore, there is a need for research about the benefits of more indirect ways to cultivate self-compassion (e.g., via a compassionate body scan) in order to improve the acceptability of self-compassion interventions in these populations.

Critically, most of the above-mentioned experimental and clinical studies did not specifically assess self-compassion (Ashar et al., 2016; Hutcherson et al., 2008; Kok et al., 2013; Weng et al., 2013). The few studies that did (Kearney et al., 2013; Neff & Germer, 2013) relied on trait-level measures that may not be sensitive to transient state changes and, like all self-report measures, may be biased by demand characteristics (Orne, 1962). Finally, these studies do not allow conclusions about the underlying mechanisms of the beneficial effects of self-compassion.

A better understanding of underlying mechanisms may come from research suggesting that compassion exerts its protective effects by stimulating physiological systems associated with stress reduction and social affiliation (Engen & Singer, 2015; Gilbert, 2009) and by reducing threat and excessive motivational drive-related arousal (Gilbert, 2009). Compassion has been positioned within the soothing and contentment system of the tripartite affect-regulation system model (Gilbert, 2009). Activating this system is proposed to enhance feeling safe, securely attached, and affiliated with others, and to enable self-soothing when stressed (Porges, 2007). It is further proposed to enhance parasympathetic activity that gives rise to the beat-to-beat variability in heart rate known as heart rate variability (HRV), which has been linked to adaptive emotion regulation in threat contexts (Thayer & Lane, 2000). This system is also suggested to promote interpersonal approach and social affiliation (Depue & Morrone-Strupinsky, 2005) mediated by activations in the central oxytocin-opiate system (Carter, 1998; Depue & Morrone-Strupinsky, 2005; Insel, 2010; Porges, 2007).

The contentment system is distinguished from a negative threat-focused affect system and from another positive affect system that is associated with stimulation and excitement, the drive system; compassion is theorized to have a downregulating effect on both (Gilbert, 2014). To date, direct support for the complete tripartite model is scarce. Kelly, Zuroff, Leybman, and Gilbert (2012) found psychometric evidence for three distinct factors—negative affect, excited positive affect, and social safeness—and for an association between low daily levels of social safeness with low levels of self-esteem and high levels of self-criticism and anxious attachment.

More indirect evidence for the model’s two positive-affect systems comes from emerging neuroscience research. First, research studying the underpinnings of compassion identified higher HRV (Arch et al., 2014; Kok et al., 2013; Rockliff, Gilbert, McEwan, Lightman, & Glover, 2008; Tang et al., 2009), reduced sympathetic activity as indicated by reduced skin conductance (Ortner, Kilner, & Zelazo, 2007; Tang et al., 2009) and lower salivary α-amylase responses (Duarte, McEwan, Barnes, Gilbert, & Maratos, 2015), reduced cortisol stress response (e.g., Rockliff et al., 2008), improved immune functioning (e.g., Davidson et al., 2003), and activation of brain circuitries associated with positive affect, compassion, and social connectedness (Klimecki et al., 2013).

Second, within a biopsychological reward model, the soothing system has been related to a behaviorally deactivating, consummatory pleasure and social engagement system, the “liking” system, whereas Gilbert’s drive system has been linked to the reward model’s “wanting” system; for example, behavior activation, seeking of reward and success, energized positive affect (Berridge & Robinson, 2016; Gray, 1987; Panksepp, 1998), and social behaviors of comparison, competitiveness, or status seeking (Bushman, Moeller, Konrath, & Crocker, 2012; Gilbert, Allan, Brough, Melley, & Miles, 2002; Sloman, Gilbert, & Hasey, 2003). Increases in incentive salience and wanting are disconnected from increases in experienced pleasure (Liggins, Pihl, Benkelfat, & Leyton, 2012) and accompanied by physiological arousal (e.g., higher heart rate; Gruber, Harvey, & Johnson, 2009) and central dopaminergic system activation (Depue & Iacono, 1989; Depue & Morrone-Strupinsky, 2005). Whereas activation of the drive system has been associated with increased positive affect and increases in self-esteem (Wood, Heimpel, & Michela, 2003), its overactivation and dysregulation have been implicated in some mental health conditions such as bipolar disorder (e.g., Johnson, McKenzie, & McMurrich, 2008). On the other hand, parasympathetic activation has been associated with the controllability of positive emotions (Kang & Gruber, 2013) and the drive system.

Additional proof for the tripartite model comes from research suggesting a role of social evaluation (Dickerson & Kemeny, 2004) and isolation (e.g., Cacioppo et al., 2000) in activating the threat system. Psychosocial stress and self-focused rumination have been associated with increased heart rate (Christian & Stoney, 2006; Woody, Smolak, Rabideau, Figueroa, & Zoccola, 2015), enhanced release of the stress hormone cortisol (e.g., Young & Nolen-Hoeksema, 2001), and augmented amygdala activation (Mandell, Siegle, Shutt, Feldmiller, & Thase, 2014). The potential benefit of activating the soothing and contentment system and of a balanced control over the threat and drive system could therefore be understood within the tripartite affect system model, but to date the role of self-compassion within this context has not been studied.

Critically, none of the above-mentioned experimental inductions were specifically designed to cultivate self-compassion. They were either based on Buddhist meditative practices incorporating mindfulness and compassion toward various different other individuals (loved ones, neutral ones, and problematic ones) after briefly directing compassion to oneself (Arch et al., 2014; Ashar et al., 2016; Fredrickson et al., 2008; Hutcherson et al., 2008; Kok et al., 2013; Weng et al., 2013), or using compassion-focused imagery, whereby participants generate an imaginary, ideal compassionate figure sending oneself unconditional love and acceptance, similar to secure attachment priming (Mikulincer & Shaver, 2007b). Although these inductions are likely to translate into greater levels of self-compassion (e.g., Kuyken et al., 2010), to date this has not been adequately tested. Furthermore, the extent to which physiological effects are specific to inducing compassionate states rather than more general positive-mood states has not been explored. In addition, the majority of the above-mentioned studies have investigated psychophysiological effects of repeated or longer-term interventions (Arch et al., 2014; Kok et al., 2013). Hutcherson et al. (2008) showed that even a brief, one-off intervention can increase social connectedness, but have not studied changes in self-compassion or physiological responses. Short-term interventions may allow experimental study of temporal dynamics of self-compassion cultivation.

Summarized, we have identified three gaps in the current literature. First, to date, experimental research into the effects and underlying mechanisms of facilitating self-compassion is lacking. Second, there is also a lack of validated, experimental, short-term self-compassion interventions and well-matched control conditions (Galante et al., 2014), and there is a particular lack of indirect self-compassion inductions for experimental research, although they have been developed for clinical practice (e.g., compassionate body scan; Neff & Germer, 2013). Third, there is a need to triangulate measures of self-compassion mechanisms by complementing self-report with physiological measures (Holmes, Craske, & Graybiel, 2014). Therefore, the aim of this study was to investigate the mechanisms whereby self-compassion confers benefits, using a novel experimental paradigm employing carefully designed experimental and control manipulations and psychophysiological measures complementing self-report.

In addition to two short self-compassion inductions to stimulate a more positive self and the affiliative affect system, we developed three control conditions in line with Gilbert’s tripartite model. As a direct technique to cultivate state self-compassion, we developed Loving-Kindness Meditation for the Self (LKM-S) with a specific focus on directing kindness and soothing to oneself (adapted from Neff & Germer, 2013). As a more indirect approach, we used a compassionate body scan (CBS) to facilitate self-compassion (adapted from Neff & Germer, 2013). We consider this a more indirect condition because participants are guided through the body, invited to attend to bodily sensations with an attitude of interest and equanimity. The teacher’s tone embodies compassion as participants are invited to recognize and allow all experiences they encounter, whether they are pleasant or unpleasant. The LKM-S practice is more explicit in inviting participants to invoke compassionate attitudes toward themselves. To maximize the integrity of the exercises, they were developed and recorded together with mindfulness teachers and eminent researchers with extensive expertise in mindfulness training.

To stimulate the drive system (Gilbert, 2009), and thus test the specificity of any effect of the self-compassion inductions, a positive-excitement condition was designed. Moreover, we included a self-critical rumination condition designed to stimulate the threat system (adapted from Roberts, Watkins, & Wills, 2013) as well as a neutral control condition (adapted from Carnelley & Rowe, 2007).

We specifically chose to recruit a nondepressed sample for this study for two reasons. First, we wanted to avoid assigning a vulnerable group to a possible distressing situation such as self-critical rumination. Second, because of clinical observation that for some people (in particular depressed individuals and self-critics) focusing on compassion for the self at first might be unfamiliar and feel unsafe (Gilbert, Baldwin, Irons, Baccus, & Palmer, 2006), we wanted to investigate the effects of our self-compassion inductions in a healthy sample first to acquire reference data before using them in a clinical sample. On the basis of previous research on compassion, we hypothesized that techniques designed to cultivate self-compassion (as compared to the control conditions) increase a more positive self and state affiliative positive affect (i.e., feeling loved and safe, feeling securely attached) and reduce negative self. It was further expected that increased self-reported positive self and affiliative affect are associated with reduced skin conductance and heart rate (inferring physiological arousal suggestive of sympathetic activation) and increased heart-rate variability (inferring increased parasympathetic activation), a physiological response pattern associated with adaptive emotion regulation. In particular, it was hypothesized that changes in the psychophysiological responses mediate the effect of self-compassion exercises on positive affiliative affect.

## Method

### Participants

We recruited a total of 135 university students in the United Kingdom (27 per experimental condition; see Fig. S1 in the Supplemental Material available online for the participant flow diagram). Participants were all students at the University of Exeter, native English speakers, right handed, with normal or corrected-to-normal vision and hearing. Exclusion criteria included current depression, currently taking psychopharmacological medication, epilepsy, cardiac problems, and a history of brain surgery. All participants provided written informed consent and received course credits or £10 for participation. The study protocol was approved by the University of Exeter School of Psychology Ethics Committee.

### Materials

#### Self-report measurements

To establish study eligibility, all participants with scores of at least 10 on the Patient Health Questionnaire for depression were excluded, as this indicates a depression diagnosis with 88% sensitivity and 88% specificity (Kroenke, Spitzer, & Williams, 2001).

To account for potential differences in trait levels of self-compassion and self-criticism, we assessed these variables across groups. We obtained a total score of the 26-item Self-Compassion Scale (SCS; Neff, 2003a), on which each item is rated on a 5-point scale ranging from 1 (*almost never*) to 5 (*almost always*), with Cronbach’s α of .69 in this sample. We determined two forms of self-criticism (inadequate self and hated self) and one form of self-reassurance using the Forms of Self-Criticizing/Attacking & Self-Reassuring Scale (FSCRS; Gilbert, Clarke, Hempel, Miles, & Irons, 2004). The FSCRS is a 22-item measure identifying different ways people think and feel about themselves when things go wrong for them; it uses a 5-point Likert scale (ranging from 0 = *not at all like me* to 4 = *extremely like me*), with Cronbach’s α in this sample of .73 for inadequate self, .76 for hated self, and .77 for reassured self.

#### Visual analogue scales for state changes

To assess the effectiveness of the experimental inductions on participants’ state self-compassion, positive affiliative affect, self-criticism, and feeling energized levels, a series of questions using visual analogue scales (VAS; ranging from 0 to 100) were used throughout the experiment (see Supplemental Material for full-prompt VAS). Three questions derived from the state adult attachment measure (Gillath, Noftle, & Stockdale, 2009) asked participants about their state affiliative affect (i.e., feeling securely attached, safe, loved, and connected; Cronbach’s α = .66 in this sample). Two questions asked about participants’ state self-compassion (Cronbach’s *r* = .73 in this sample), adopted from the SCS (Neff, 2003a), one about their state self-criticism (based on the FSCRS; Gilbert et al., 2004), and one about how energized they felt.

#### Experimental inductions

The induction tapes for the five different conditions were developed and recorded together with an experienced MBCT therapist who had been trained in MBCT and taught > 10 courses. The tapes were matched in terms of length (11.5 min) and word density (610–630 words). Instructions were evenly distributed throughout the experimental inductions. In the CBS, participants were guided to direct kind and compassionate attention to their body sensations, starting from the top of the head and going down to the feet. In the LKM-S condition, participants were first guided to bring to mind a person they felt a natural sense of warmth toward and to direct friendly wishes toward this person. After this, participants were invited to offer the same friendly wishes toward themselves. In the self-critical rumination condition, participants were asked to dwell on something they felt they had not managed or achieved as they would have wanted to. In the control condition, participants were guided through a routine supermarket shopping scenario. In the positive excitement condition, participants were asked to think about certain aspects of a positive event or situation in which they were working through or achieving something great. Feedback on the final audio exercises was gathered from experienced mindfulness and meditation practitioners, as well as staff within our clinical department, to ensure ecological validity.

### Psychophysiological recording and data preprocessing

The autonomic nervous system measures described below were recorded using a BIOPAC MP150 system and the software AcqKnowledge 4.2 (BIOPAC Systems; Goleta, CA), with an acquisition sampling rate of 2000Hz. After recording, these data were processed using specialized analysis programs within the AcqKnowledge 4.2 software as described in the respective sections below.

#### Heart rate

Heart rate (HR) was acquired as an indicator of physiological arousal. HR was determined from raw electrocardiography (ECG) in beats per min on the basis of a semiautomatic R-wave detection algorithm implemented in the software AcqKnowledge (version 4.2., BIOPAC Systems Inc., Goleta, CA). Raw ECG was acquired using a BIOPAC ECG100C amplifier at a sampling rate of 1 kHz and filtered using a band pass of 0.5–35 Hz. Artifact detection (i.e., noisy, missing, or ectopic beats) and removal was performed using a template correlation and interpolation from the adjacent R-peaks based on established procedures (Berntson, Quigley, Jang, & Boysen, 1990). The interpolation procedure was used for less than 5% of the ECG data. Mean HR in beats per min was then extracted from the R-waves for each data section.

#### High-frequency heart-rate variability

High-frequency heart-rate variability (HF-HRV) as an indicator of parasympathetic activation and adaptive physiological regulation capacity (Thayer & Lane, 2000) was determined from the artefact-free ECG (see above) by submitting a time series of the R-peaks to a fast Fourier transformation that calculated the power spectrum of the R-R interval variation for the frequency range between 0.15 Hz and 0.4 Hz in a given time window (Berntson et al., 1997). Mean HF HRV were then extracted for each data section similar to the heart rate. HRV values were log-transformed using the natural log to normalize data.

#### Skin conductance level

Skin conductance was applied as a measure of sympathetic activation and physiological defense response (Sokolov, 1963). It was continuously recorded using a BIOPAC SCL100C amplifier and a skin-resistant transducer (TSD203) from the middle phalanx of the first and ring fingers of the participant’s nondominant hand at a sampling rate of 500 Hz with a low pass filter of 1.0 Hz. Mean skin conductance level (SCL), maximum SCL values, and minimum SCL values were extracted for the same time windows and a range correction (Lykken, Rose, Luther, & Maley, 1966) was applied to each data section for each participant to give a mean SCL corrected for individual differences. The formula for this was Corrected SCL = (SCLmean – SCL min)/(SCL max – SCL min).

To obtain measures of HR, HRV, and SCL change throughout the audio exercise and in order to control for individual differences, we calculated participants’ change values for each minute of the experimental condition by subtracting the participants’ averaged baseline value from the value for each subsequent 1-min section of the audio exercise. The last 30 s of each recording were excluded from the analyses because all experimental inductions lasted less than 11.5 min, and a recording of approximately 1 min is needed to reliably assess the physiological components analyzed in this study (Berntson et al., 1997).

### Procedure

We screened participants for the exclusion criteria using an online survey. Eligible participants were invited to the laboratory session. Following informed consent, participants completed a self-referential task (repeated at the end but not reported here). Participants then completed an 8-min baseline (divided into eight, 1-min blocks—four with their eyes open and four with their eyes closed—for an electroencephalography study not reported here) in which subjects were invited to relax. Next, participants listened to one of the five induction tapes and finally were asked to complete a 1-min resting period with their eyes closed (the analyses of the postinduction findings are reported in the Supplemental Material). Before and after the first baseline and following the induction, participants completed a manipulation check using visual analogue scales as described above. During the whole experimental procedure, ECG and SCL were continuously recorded.

### Statistical data analysis

Data were analyzed using IBM SPSS Statistics (Version 21), R (http://www.r-project.org), and Mplus (Version 7.3; Muthén & Muthén, 2014).

#### Manipulation checks

For testing the effectiveness of the experimental inductions on participants’ state self-compassion, positive affiliative affect, and self-criticism, a series of repeated measures analyses of variance (ANOVAs) were conducted, with time (pre- vs. postmanipulation) as the within-subjects factor and condition as the between-subjects factor.

#### Latent growth curve modelling

To investigate if the different experimental inductions were associated with different physiological response trajectories throughout the task, a latent growth curve modeling (LGCM) approach was applied using MPlus software (Muthén & Muthén, 2014). LGCM is a novel statistical approach for longitudinal and repeated measures data that combines and extends features of repeated measures ANOVA and structural equation modeling (Duncan, Duncan, & Strycker, 2011) and allows the capture of the average trend or pattern of change over time and between-person differences around the average trend (Browne, 1993; Meredith & Tisak, 1990; Muthén & Curran, 1997; Willett & Sayer, 1994).

LGCM fits a basic growth model in which repeated measures of a variable represent indicators of continuous latent variables—growth factors—that represent different aspects of change and capture individual differences in a trajectory (Meredith & Tisak, 1990). Typically, these are the intercept (i.e., mean starting value) and the linear (i.e., rate of growth) and quadratic (i.e., leveling off, or coming down) slopes. We initially centered the intercept at Minute 1 of the exercises. In order to understand the role of the different experimental conditions, we added dummy-coded variables CBS, LKM-S, Rumination, and Positive Excited conditions (thus running it against the neutral condition) as covariates to our growth curve model. The resulting coefficients therefore signify the contribution of each respective condition in the context of all other conditions; for example, whether each condition differed significantly from the neutral condition (which was expected to reveal no significant change). In addition to centering the intercept at Minute 1 of the exercises, we ran models with different center points from Minutes 2 to 11 to describe the influence of our conditions at different times during the exercises. We followed the suggested procedures of Muthén and Muthén (2000), who stated that models with varying centering points are reparameterizations of each other. Analysis will therefore result in the same model fit and is superior to regular regressions, as it draws on information from all time points.

Model fit was determined using root-mean-square approximation (RMSEA), comparative fit index (CFI), the Tucker-Lewis index (TLI), and the standardized root-mean-square residual (SRMR; see Schermelleh-Engel, Moosbrugger, & Mueller, 2003). Comparisons between the different models within each outcome variable were made informal based on the sample size adjusted Bayesian Information Criterion (aBIC), the Akaike Information Criterion (AIC), whereby smaller values indicate a better model fit.

#### Correlational analyses

In order to study the associations between experimental condition, psychophysiological responses, and state changes in self-report, we first calculated zero order correlations (Pearson) using SPSS. Residualized gain scores, as validated index of pre-post change that controls for variance in initial prescores, were calculated for each person by regression with postscore as outcome (Mintz, Luborsky, & Christoph, 1979; Williams, Zimmerman, Rich, & Steed, 1984). Physiological change values as index of overall physiological change were calculated by averaging participants’ change values for each minute of the experimental condition together into a single variable. In order to study the associations between the experimental conditions, physiological responses, and state changes in self-report, we used dummy-coded variables for the experimental conditions CBS, LKM-S, Rumination, and Positive Excitement that were each contrasted against the neutral condition.

Using Mplus, we then calculated a series of simple mediations with self-reported state change as outcome, experimental condition as predictor, and physiological response as mediator. To determine the size of direct and indirect effects, we followed principles suggested by Hayes (2012), and to adjust for smaller samples we used bias corrected confidence intervals (Efron & Tibshirani, 1993; Fritz & MacKinnon, 2007). To account for multiple testing, we adjusted the *p* value for number of tests.

### Data and material availability

All data, code, and material are available from the authors on reasonable request.

## Results

### Sample characteristics

Sample characteristics are shown in Table 1. The average age of the sample was 19.34 years (*SD* = 2.06). Trait levels of self-compassion in this sample were similar to published self-compassion scores for healthy young adults (*M* = 19.51 out of 30, *SD* = 4.46, range = 8.60– 28.90 as compared to *M* = 18.25, *SD* = 3.75; Neff, 2003a). Furthermore, participants in this study can be described as relative low in self-criticism as compared to previously published self-criticism scores for nonclinical populations (“inadequate self” subscale of the FSCRS: *M* = 12.97 out of 36, *SD* = 7.27, range = 0.00–33.00 vs. *M* = 17.72, *SD* = 8.29; Baiao, Gilbert, McEwan, & Carvalho, 2015). As shown in Table 1, there were no significant differences between the groups in age, levels of self-compassion (SCS), and levels of self-criticism (FSCRS). Importantly, the different groups were comparable in terms of their self-reported state levels of self-compassion (*F*(4,130) = 0.64, *p* = .637, η_*p*_^2^ = .02), self-criticism (*F*(4,130) = 0.35, *p* = .845, η_*p*_^2^ = .01), positive affiliative affect (*F*(4,130) < .40, *p* = .809, η_*p*_^2^ = .01), and feeling energized (*F*(4,130) = 1.06, *p* = .380, η_*p*_^2^ = .03). In addition, no significant group differences emerged for the physiological parameters at baseline (see Table 1).

**Table 1.** Sample Characteristics

	Group							
Characteristic	LKM-S	CBS	Rumination	Positive Condition	Neutral Condition	Test	*p*	η_*p*_^2^
*n*	27	27	27	27	27			
Gender: male/female (*n*)	7/20	7/20	7/20	7/20	7/20			
Age in years: *M* (*SD*)	18.81 (1.36)	19.81 (2.83)	19.60 (2.30)	18.93 (1.41)	19.50 (1.88)	*F*(4, 134) = 1.35	.254	0.04
Self-Compassion Scale total sum: *M* (*SD*)	19.75 (5.11)	20.16 (4.84)	18.61 (3.62)	19.83 (4.23)	19.19 (4.51)	*F*(4, 134) = 0.58	.673	0.02
FSCRS								
Reassure self: *M* (*SD*)	21.25 (5.53)	21.70 (5.11)	19.85 (5.66)	20.96 (5.94)	19.44 (5.53)	*F*(4, 134) = 0.79	.528	0.02
Inadequate self: *M* (*SD*)	13.05 (7.27)	11.70 (6.86)	14.48 (8.17)	12.41 (6.63)	13.22 (7.26)	*F*(4, 134) = 0.56	.692	0.01
Hated self: *M* (*SD*)	1.59 (3.24)	1.26 (1.74)	1.88 (2.66)	1.22 (1.50)	2.77 (3.26)	*F*(4, 134) = 1.63	.171	0.04
Physiological baseline measures								
Skin-conductance level in microSiemens: *M* (*SD*)	0.33 (0.22)	0.44 (0.25)	0.39 (0.25)	0.47 (0.23)	0.44 (0.25)	*F*(4,126) = 1.45	.221	0.04
Heart rate in BPM: *M* (*SD*)	74.89 (8.73)	77.38 (7.63)	74.80 (9.72)	70.56 (11.81)	71.78 (7.83)	*F*(4,126) = 2.27	.065	0.07
Heart rate variability in ms^2^/Hz: *M* (*SD*)	4.49 (1.07)	5.04 (0.95)	4.83 (1.53)	5.10 (1.04)	5.02 (1.41)	*F*(4,126) = 1.05	.382	0.03
Physiological baseline measures after exercise								
Skin conductance level in microSiemens: *M* (*SD*)	0.41 (0.26)	0.45 (0.26)	0.40 (0.23)	0.49 (0.19)	0.47 (0.22)	*F*(4,107) = 0.44*	.777	0.01
Heart rate in BPM: *M* (*SD*)	72.51 (11.05)	75.71 (8.20)	69.94 (16.75)	70.93 (11.92)	71.09 (9.55)	*F*(4,109) = 0.72*	.578	0.03
Heart rate variability in ms^2^/Hz: *M* (*SD*)	4.70 (0.93)	5.30 (1.03)	4.43 (1.35)	4.97 (1.08)	4.79 (1.00)	*F*(4,101) = 1.54*	.195	0.06

Note: LKM-S = Loving-Kindness Meditation for the Self; CBS = compassionate body scan; FSCRS = Forms of Self-Criticizing/Attacking & Self-Reassuring Scale; BPM = beats per min.

### Manipulation checks

#### Changes in state self-compassion

**Fig. 1.**
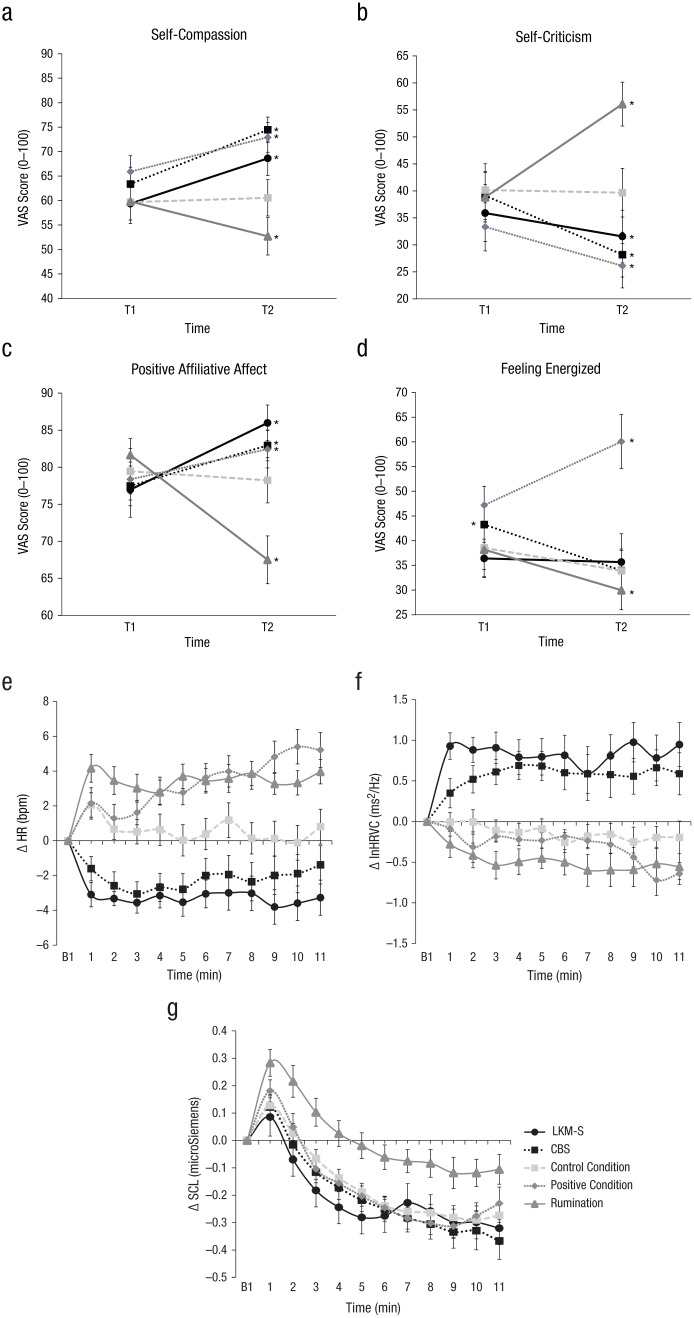
Psychophysiological results for (a) changes in state self-compassion, (b) changes in state self-criticism, (c) state positive affiliative affect, (d) changes in feeling energized, (e) baseline-to-exercise change in heart rate (HR), (f) baseline-to-exercise change in heart-rate variability change (HRVC), and (g) baseline-to-exercise change in skin conductance level (SCL). Asterisks indicate significant change between the preexperimental manipulation (T1) and postexperimental manipulation (T2). Baseline scores for the physiological responses have been set to zero (e.g., we deducted the mean baseline activity from the activity during the exercise). Growth models were run on response scores/baseline-corrected scores. Error bars indicate ± 1 *SE*. bpm = beats per minute; LKM-S = Loving-Kindness Meditation for the Self; CBS, Compassionate Body Scan.

The scores for the state self-compassion ratings are depicted in Figure 1a. The Group × Time ANOVA revealed a main effect of Group, *F*(4, 130) = 2.86, *p* = .026, η_*p*_^2^ = .08, which, in line with our hypothesis, was qualified by a significant Group × Time interaction, *F*(4,130) = 12.65, *p* < .001, η_*p*_^2^ = .28. Post hoc analyses revealed that there was a significant increase in self-compassion in the CBS condition, *F*(1, 26) = 27.56, *p* < .001, η_*p*_^2^ = .51, 95% confidence interval (CI) = [6.73, 15.41], and for the LKM-S condition, *F*(1, 26) = 23.30, *p* < .001, η_*p*_^2^ = .47, 95% CI = [5.32, 13.20]. A similar but smaller effect could be found for the positive condition, *F*(1, 26) = 12.63, *p* = .001, η_*p*_^2^ = .37, 95% CI = [2.96, 11.07]. In contrast, a significant decrease in self-compassion could be found in the rumination condition, *F*(1, 26) = 7.47, *p* = .011, η_*p*_^2^= .22, 95% CI = [–12.42, –1.76]. There was no pre-to-post difference in the control condition, *F*(1, 26) = .27, *p* = .607, η*_p_*^2^ = .01, 95% CI = [–2.61, 4.96]. Interestingly, an ANCOVA on postinduction scores (see Supplemental Materials) using pre-induction scores as the covariate revealed that after induction only individuals in the two self-compassion conditions (but not those in the positive excited condition) reported significantly higher self-compassion than the neutral condition, and individuals in the rumination condition reported significantly lower self-compassion.

#### Changes in state self-criticism

Similarly, the Group × Time ANOVA yielded a main effect of Group, *F*(4,130) = 2.64, *p* = .037, η_*p*_^2^ = .08, which again was qualified by a significant time by group interaction, *F*(4, 130) = 12.33, *p* < .001, η_*p*_^2^ = .28. The scores for the state self-criticism ratings are depicted in Figure 1b. Post hoc analyses revealed that there was a significant pre-to-post decrease in self-critical ratings in the CBS group, *F*(1, 26) = 8.55, *p* = .006, η_*p*_^2^ = .25, 95% CI = [–18.46, –3.66] and for the LKM-S condition, *F*(1, 26) = 7.00, *p* = .014, η_*p*_^2^ = .21, 95% CI = [–7.69, 0.97]. A similar but smaller effect was found for the positive condition, *F*(1, 26) = 7.54, *p* = .044, η_*p*_^2^ = .15, 95% CI = [–14.23, 0.22]. In contrast, there was a significant increase in self-critical ratings in the rumination condition, *F*(1, 26) = 21.11, *p* < .001, η_*p*_^2^ = .45, 95% CI = [9.54, 24.98]. No pre-to-post manipulation difference emerged for the control condition, *F*(1, 26) = .03, *p* = .857, η_*p*_^2^ < .00, 95% CI = [–5.93, 4.96]. Interestingly, an ANCOVA (see Supplemental Material) revealed that after induction, only individuals in the rumination condition reported significantly higher state levels of self-criticism as compared to the neutral condition.

#### Changes in state positive affiliative affect

The scores for the positive affiliative affect ratings are depicted in Figure 1c. The Group × Time ANOVA revealed no significant main effect of group, *F*(4,130) = 1.03, *p* > .05, η_*p*_^2^= .03. However, the Time × Group interaction yielded significance, *F*(4, 130) = 24.46, *p* < .001, η_*p*_^2^= .43. Post hoc analyses revealed that there was a significant pre-to-post increase in positive affiliative affect in the CBS condition, *F*(1, 26) = 10.53, *p* = .003, η_*p*_^2^ = .28, 95% CI = [2.00, 8.93], the LKM-S condition, *F*(1, 26) = 26.79, *p* < .001, η_*p*_^2^= .51, 95% CI = [5.43, 12.59] and, albeit smaller, for the positive condition, *F*(1, 26) = 6.12, *p* = .020, η_*p*_^2^= .19, 95% CI = [0.69, 7.46]. In the rumination condition there was a significant decrease in positive affiliative affect after the manipulation, *F*(1, 26) = 38.90, *p* < .001, η_*p*_^2^= .60, 95% CI = [–18.79, –9.48], whereas no pre-to-post manipulation difference emerged for the control condition, *F*(1, 26) = .49, *p* = 486, η_*p*_^2^= .01, 95% CI = [–4.77, 2.33]. Interestingly, an ANCOVA (see Supplemental Material) revealed that after induction, only individuals in the LKM-S condition reported significantly higher positive affiliative affect than those in the neutral condition, and individuals in the rumination condition reported significantly lower positive affiliative affect.

#### Changes in feeling energized

The scores for the feeling energized ratings are depicted in Figure 1d. The Group × Time ANOVA revealed a significant main effect of group, *F*(4,130) = 3.63, *p* = .008, η_*p*_^2^ = .01, that was qualified by the significant Time × Group interaction, *F*(4, 130) = 6.24, *p* < .001, η_*p*_^2^ = .16. Post hoc analyses revealed that there was a significant pre-to-post increase in feeling energized in the positive excitement condition, *F*(1, 26) = 11.15, *p* = .003, η_*p*_^2^ = .30, 95% CI = [4.95, 20.82]. In contrast, there was significant decrease in feeling energized for the CBS condition, *F*(1, 26) = 6.10, *p* = .021, η_*p*_^2^ = .19, 95% CI = [–17.04, –1.55] and for the rumination condition, *F*(1, 26) = 4.68, *p* = .040, η_*p*_^2^= .15, 95% CI = [–16.03. 0.41]. No pre-to-post manipulation difference emerged for the control condition, *F*(1, 26) = 3.27, *p* = .082, η_*p*_^2^= .11, 95% CI = [–9.97, 0.64], or the LKM-S condition, *F*(1, 26) = .04, *p* = .847, η_*p*_^2^ = .04, 95% CI = [–8.53, 7.05].

ANCOVAs on postinduction scores, using pre-induction scores as the covariate, revealed that only individuals in the positive excited condition showed elevated feeling energized at the end of the exercise (see Supplemental Material).

### Effects of the self-compassion and control manipulations on physiological responses

#### Heart-rate effects

Figure 1f shows the pattern of change in heart rate for the different experimental conditions. The outcome variables were multivariately normally distributed. The model with continuous latent variables of intercept of heart-rate change at Minute 1 and linear and quadratic slope as outcome and the five experimental conditions as independent variables revealed a good fit with *χ*^2^(89) = 164.66, *p* < .001; CFI = .968; TLI = .965; SRMR = .03; RMSEA = .08, 90% CI = [0.06, 0.09]; AIC = 6,648.53; aBIC = 6,639.80. It indicated that the CBS (*b* = −3.66, *SE* = .99, *p* < .001), Rumination (*b* = 2.32, *SE* = 1.00, *p* = .020), and LKM-S (*b* = −4.54, *SE* = .99, *p* < .001) were significantly influencing the intercept, but there were no linear or quadratic slope effects for these conditions (all *p* > .05). This suggests that relative to the neutral condition, heart rate was decreased at the start of the CBS and the LKM-S, whereas it was elevated in the rumination condition. In contrast, the positive excitement condition had a significant effect on the linear slope (*b* = 0.825, *SE* = .33, *p* = .012), suggesting that heart rate consistently increased over the course of this intervention. Additional models recentering the intercept revealed that whereas being in LKM-S and Rumination conditions was significantly associated with heart rate at all 11 min, being in the CBS condition ceased to be significant in Minute 8 and being in the positive condition made a significant contribution from Minute 4 through Minute 11.

#### Heart-rate variability effects

Figure 1g depicts the pattern of change in heart-rate variability for the different experimental conditions. As the outcome variables were not multivariate normally distributed, we used the maximum likelihood estimation with robust standard errors (MLR). The model with continuous latent variables of intercept at Minute 1, slope, and quadratic growth of heart-rate variability as outcome and the five experimental conditions as independent variables revealed a good fit with *χ*^2^(89) = 176.83, *p* < .001, CFI = .943; TLI = .936; SRMR = .03; RMSEA = .08, 90% CI = [0.068, 0.105]; AIC = 2,145.70; aBIC = 2,136.50. The LKM-S (*b* = 0.91, *SE* = 0.18, *p* < .001) and the rumination condition (*b* = −0.39, *SE* = 0.10, *p* = .035) had a significant effect on the intercept at Minute 1 but there were no linear or quadratic effects, indicating that HRV was elevated at the start of the LKM-S and decreased at the start of the rumination condition relative to the neutral condition. The CBS not only had a significant effect on the intercept of HRV (*b* = 0.40, *SE* = .17, *p* = .022), but also a significant linear (*b* = 0.14, *SE* = .05, p = .013) and quadratic growth effect (*b* = −0.01, *SE* < 0.01, *p* = .019). These results suggest that the HRV was elevated at the start of the CBS relative to the neutral condition and increased slowly over the first few minutes, plateaued at Minute 4, and then decreased again at the last minute of the intervention. Additional models recentering the intercept yielded that being in the two self-compassion conditions, CBS and LKM-S, was significantly associated with HRV intercept at all 11 min. Being in the rumination condition ceased to be significant in Minute 5, and being in the positive condition started to make a significant contribution in Minute 10.

#### Skin-conductance level effects

The skin-conductance level results are depicted in Figure 1g. As the outcome variables were not multivariate normally distributed, we used the MLR. A piecewise model with continuous latent variables of intercept, one linear and quadratic slope of skin-conductance change from Minute 1 to Minute 7, and a second linear slope from 8 to 11 as outcomes, and the five experimental conditions as independent variables revealed a partially acceptable fit with χ^2^(80) = 266.81, *p* < .001, CFI = .915; TLI = .895; SRMR = .030; RMSEA = .132, 90% CI = [0.114, 0.149]; AIC = −3,082.63; aBIC = −3,093.21. Only the rumination condition had a significant effect on the intercept at Minute 1 (*b* = 0.16, *SE* = 0.06, *p* = .005) but no other significant effects. This finding suggests that skin-conductance level was elevated at the start of the rumination condition as compared to the neutral condition. Moreover, during Minute 1 to Minute 7, the LKM-S had a significant effect on the linear (*b* = −0.04, *SE* = 0.02, *p* = .038) and quadratic slope (*b* = 0.01, *SE* = 0.003, *p* < .001), indicating that relative to the neutral condition, skin-conductance level decreased more steeply in this experimental condition but also moved up toward the end of the first 7 min. There were no significant slope effects for Minute 8 to Minute 11. Additional models recentering the intercept revealed that being in the rumination condition was significantly associated with SCL at all 11 min. Being in the LKM-S condition made a significant contribution to the intercept growth factor between Minutes 2 and 5.

### Associations between self-compassion condition, psychophysiological response changes, and changes in self-reported positive affiliative affect

Table 2 shows the zero order correlations. Both self-compassion conditions were significantly correlated with HR reduction and HRV increase, as well as with self-reported increase in self-compassion and positive affiliative affect. In contrast, the opposite pattern of significant correlations was found for the rumination condition. Being in the positive condition was significantly correlated with change in self-reported self-criticism and feeling energized but not change in self-compassion or positive affiliative affect. More interestingly, it was significantly associated with similar physiological response changes as in the rumination condition; for example, increased HR and reduced HRV. Feeling energized was significantly negatively correlated with the rumination condition and the CBS. The Supplemental Material includes a detailed consideration of effect sizes and differences between correlation coefficients.

**Table 2.** Summary of Key Correlation Findings

	Key correlations						
	State self-report change				Psychophysiological change		
Measure	∆ Self-compassion	∆ Self-criticism	∆ PAA	∆ FeelEn	∆ HR	∆ HRV	∆ SCL
Psychophysiological change							
∆ Heart rate	−.176*	.239**	−.266**	.155			
∆ Heart rate variability	.159	−.236**	.246**	−.106			
∆ SCL	−.182*	.287**	−.349**	.044			
Experimental conditions							
LKM-S	.192*	−.104	.344**	.012	−.381**	.392**	−.179*
CBS	.303**	−.267**	.195*	−.174*	−.275**	.264**	−.156
Rumination	−.492**	.538**	−.612**	−.171*	.322**	−.314**	.458**
Pos Con	.154	−.207*	.146	.411**	.312**	−.223*	−.057
Control	−.157	.040	−.073	−.073	.016	−.121	−.067

Note: PAA = positive affiliative affect; FeelEn = feeling energized; HR = heart rate; HRV = heart rate variability; SCL = skin conductance level; LKM-S = Loving-Kindness Meditation for the Self; CBS = compassionate body scan; Pos Con = positive condition.

* Correlation is significant at the 0.05 level (2-tailed). **Correlation is significant at the 0.01 level (2-tailed).

Overall, the effects were small to medium, and HR change emerged as the only parameter that was significantly correlated with all predictor and outcome variables; therefore, mediation analyses were performed for this parameter to investigate if changes in HR precede changes in self-reported changes in both self-compassion conditions. In the Supplemental Material, we report findings for the other conditions.

### Heart-rate change as mediator for effects of self-compassion inductions on self-report

#### Change in state self-compassion

Neither for the LKM-S nor for the CBS condition was there evidence of a significant mediation effect of change in HR.

#### Change in state self-criticism

**Fig. 2.**
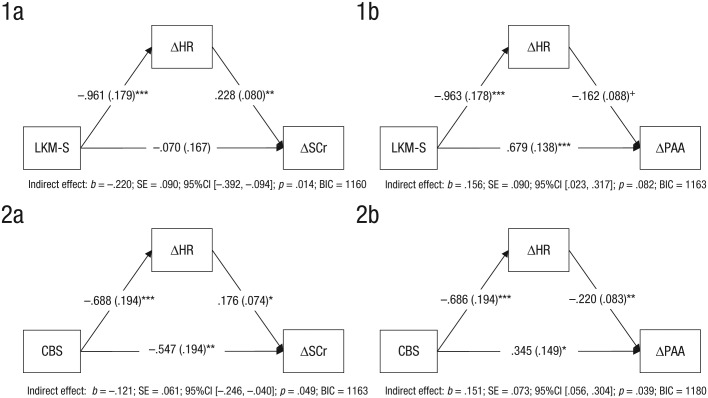
Mediation analyses for HR responses as mediators for the effect of the LKM (Panels 1A and 1B) and the CBS (Panels 2A and 2B) on changes in self-report. HR = heart rate; SCr = self-criticism; PAA = positive affiliative affect; LKM-S = Loving Kindness Meditation for the Self; CBS = Compassionate Body Scan.

For the CBS condition, there were again significant direct and indirect effects (see Figure 2, panel 2A), suggesting a partial mediation; for example, that the body scan exerts its effect on decreasing self-criticism directly and via a reduction in HR. For the LKM-S, the direct path was not significant but a significant indirect effect was identified, suggesting a full mediation; for example, that LKM-S exerted its effect on reducing self-criticism only via a reduction in HR (see Figure 2, Panel 1A).

#### Change in state positive affiliative affect

For the CBS condition, there were significant direct and indirect effects (see Figure 2, Panel 2B), suggesting a partial mediation; for example, that the body scan exerts its effect on increasing positive affiliative affect directly and via a reduction in HR. Although the effects were in a similar direction for LKM-S, they failed to reach significance (see Figure 2, Panel 1B).

#### Change in feeling energized

Neither for the LKM-S condition nor the CBS condition were there significant mediation effects of HR.

## Discussion

In this study we used two short-term experimental inductions designed to temporarily increase self-compassion, as well as control conditions stimulating either the threat or the drive systems, to test the hypothesis that both a CBS and an LKM-S, as compared to the control conditions, reduce state self-criticism and physiological arousal on one hand and increase state positive affiliative affect, self-compassion, and parasympathetic activation on the other hand. Furthermore, we investigated whether changes in the psychophysiological responses mediate the effect of self-compassion exercises on state changes in self-compassion, self-criticism, and positive affiliative affect.

The results were largely in line with our expectations and lead us to suggest that self-compassion may exert its beneficial effects on mental and physical health in two possible ways, first, by temporarily activating a low-arousal parasympathetic positive affective system that has been associated with stress reduction, social affiliation, and effective emotion regulation, and second, by temporarily increasing positive self and reducing negative self, thus addressing cognitive vulnerabilities for mental health problems such as depression. Integrating our findings into the existing literature, we discuss this before providing a discussion of the relevance of our findings for the tripartite model of emotion regulation and the wider theoretical implication for the construct of self-compassion.

### Short-term self-compassion exercises may exert their beneficial effect by temporarily activating a low-arousal parasympathetic positive affective system that has been associated with stress reduction, social affiliation, and effective emotion regulation

In a sample of healthy individuals, both the LKM-S and the CBS led to sustained increases in parasympathetic activity indicated by higher HRV over 11 min, and sustained decreases in physiological arousal indicated by paralleled decreases in HR and lower skin-conductance levels. This physiological state has been described earlier as hypometabolic (Benson, Beary, & Carol, 1974) or low-arousal parasympathetic state. It has been associated with health benefits (Kanji, White, & Ernst, 2006) and can be elicited by a number of interventions, such as relaxation exercises (e.g., Kanji et al., 2006; King, 1980; Lim & Kim, 2014; Pawlow & Jones, 2005), mindfulness (e.g., Krygier et al., 2013; Zeidan, Johnson, Gordon, & Goolkasian, 2010), and hypnosis (Yuksel, Ozcan, & Dane, 2013). It has also been observed after activating a mental representation of a secure attachment figure (Bryant & Hutanamon, 2018). More importantly, this state has also previously been associated with the cultivation of compassion, as indicated by increased parasympathetic activity (Arch et al., 2014; Kok et al., 2013; Rockliff et al., 2008), decreased physiological arousal (Tang et al., 2009), reduced cortisol levels (Rockliff et al., 2008), and improved immune functioning (Breines et al., 2014).

Our results are thus in line with these previous findings and extend them in several ways. First, we showed that in a nonthreatening context, both a more direct (LKM-S) and a more indirect (CBS) self-compassion exercise not only induce a relaxed physiological state previously associated with health benefits but also stimulate the soothing and contentment system previously linked to healthy tolerance for distress, and a motivation to care for oneself and others (Gilbert, 2009; Gillath, Shaver, & Mikulincer, 2005). Both exercises induced higher parasympathetic activity, as indicated by increased HRV. Higher HRV has been associated with more effective emotion regulation (Appelhans & Luecken, 2006), efficient moment-to-moment cognitive processing of affective information (Hildebrandt, McCall, Engen, & Singer, 2016), and physical and psychological health (Appelhans & Luecken, 2006; Thayer & Lane, 2000, 2007). Moreover, higher HRV has been suggested to be conducive to social affiliation and the ability to self-soothe when stressed (Depue & Morrone-Strupinsky, 2005; Porges, 2007). This ability is developmentally shaped by early experiences with a caregiver and throughout life; Healthy social relationships are important for dealing with aversive life events using successful emotion regulation (Mikulincer & Shaver, 2007a) and for facilitating cardiovascular health (Cacioppo et al., 2000). Our finding of higher positive affiliative affect induced by the two self-compassion conditions is also in line with research in which short-term (Hutcherson et al., 2008) and long-term compassion training (e.g., Klimecki et al., 2013; Kok et al., 2013) have been shown to increase social connectedness. Furthermore, in line with Depue and Morrone-Strupinsky (2005) and Gilbert (2014), who argued that the stimulation of the soothing and contentment system is associated with down-regulation of the threat and drive system, in this study, the self-compassion inductions, but not the positive control condition, led to reduced physiological arousal, as indicated by sustained reductions in HR and skin conductance.

Second, by looking at the psychophysiological response trajectories, we were able to study the time course of physiological changes as they unfolded during the self-compassion exercises. This allowed us to identify subtle differences between LKM-S and CBS. The LKM-S condition was characterized by sustained HRV increase over the entire time, whereas the CBS led to a slowly rising HRV that reached its plateau a few minutes into the exercise before it was sustained over several minutes. This indicates that the HRV trajectory was affected slightly differently in a more direct versus indirect self-compassion induction, but further research is necessary to fully understand why CBS took longer to reach the HRV peak. One possible explanation for subtle differences between the LKM-S and CBS might be the somatic versus nonsomatic focus of the self-compassion exercises. In addition, considering the response trajectories also allowed us to identify an interesting pattern of gradual increase in HR over time to the positive excited condition that is further discussed below. Interestingly, analyses of the physiological postinduction baseline measurements revealed no significant group differences (see Supplemental Material). This indicates that the physiological changes associated with the different experimental conditions were not sustained outside the exercises. In contrast to previous research that compared baseline HRV (Arch et al., 2014; Kok et al., 2013), this was a one-off short-term audio exercise unlikely to change baseline (trait-level) physiological activity. Although Kok et al. (2013) found increases in baseline HRV after a 6-week compassion intervention, existing literature on changes in trait-level physiological measures is mixed to date, with Arch et al. (2014) not finding such changes. An interesting avenue for further research might be to look at baseline changes within long-term interventions that have been reported to lead to a significant increase in trait-level self-compassion, such as MBCT (Kuyken et al., 2010) or Compassion Focused Therapy (Gilbert, 2014).

Third, by showing that physiological responses to both self-compassion conditions were significantly associated with the extent of changes in self-report, suggesting that higher HR reduction and HRV increase are associated with higher increases in self-compassion and positive affiliative affect, whereas the opposite associations were found for changes in self-criticism. Moreover, change in HR mediated effects of LKM-S and CBS on state self-criticism and partially on state positive affiliative affect. This finding suggests that self-compassion exercises may soothe the heart, which in turn may lead to the experience of feeling safe and connected with others. However, a reversed dynamic in which the experience (self-report) precedes the HR reduction cannot unequivocally be ruled out because, for methodological reasons, we did not ask for self-report ratings during the exercise. Thus, further research is necessary to understand the temporal dynamics of physiological and self-report change. One possible avenue is to complement the study of physiological response trajectories with an in-depth study of the unfolding of the experience by applying qualitative neurophenomenological approaches (Olivares, Vargas, Fuentes, Martinez-Pernia, & Canales-Johnson, 2015).

### Short-term self-compassion exercises may exert their beneficial effect by temporarily increasing positive self and reducing negative self-bias, thus potentially addressing cognitive vulnerabilities for mental disorders

Critically, the results of this study are extending previous research by directly inducing self-compassion and by assessing state changes in self-compassion, self-criticism, and positive affiliative affect. Both self-compassion conditions temporarily activated a more positive (self-compassion) and less negative self (self-criticism). This is in line with Falconer et al. (2014), who reported state improvement in self-compassion and self-criticism after compassion-focused virtual reality. Stable and accepting positive self has previously been associated with higher levels of well-being, whereas pervasive negative self-bias is at the core of a number of mental health problems (Cili & Stopa, 2015; Mezulis, Abramson, Hyde, & Hankin, 2004). For example, the cognitive vulnerability model for depression highlights that the disorder is maintained by a pervasive negative self-bias; for example, increased automatic access to negative information about the self that is difficult to inhibit, while at the same time positive information about the self is difficult to retrieve (Segal et al., 2013). Inviting individuals to direct compassion toward themselves (at their body, as in CBS, or at the metarepresentation of their self, as in LKM-S) may thus also work for addressing cognitive vulnerabilities in self-referential processing. Notably, our LKM-S condition differed from that used in previous research (Arch et al., 2014; Ashar et al., 2016; Fredrickson et al., 2008; Hutcherson et al., 2008; Kok et al., 2013; Weng et al., 2013) by inviting participants to direct compassion to a secure attachment figure/loved one first, and then to direct compassion to the self for several minutes. In support of a possible role of self-compassion for addressing cognitive vulnerability, we have recently shown that individuals with a history of recurrent depression who reported greater self-compassion showed lower self-devaluation and more efficient mood recovery from a sad mood induction (Karl, Williams, Cardy, Kuyken, & Crane, 2018). That the activation of the above physiological pattern partially preceded increased positive and reduced negative self indicates that a state of low physiological arousal (calm and content) in a nonthreatening and nondrive situation may enable openness for altered cognitive appraisals (Fredrickson et al., 2008), but this should be more directly investigated; for example, by using self-referential tasks (Markus, 1977).

### An experimental paradigm to test self-compassion within the tripartite model of emotion regulation: theoretical considerations

The inclusion of carefully designed control conditions further allowed us to fully test Gilbert’s (2009) tripartite model of emotion regulation. We have previously discussed how the two self-compassion conditions have been shown to activate a psychophysiological response pattern indicative of the soothing system.

Because of its self-critical and socially evaluative nature, the rumination condition effectively stimulated the threat-focused affect system (Dickerson & Kemeny, 2004; Gilbert, 2009). Specifically, this condition was associated with decreased self-reported levels of state self-compassion and positive affiliative affect, as well as increases in state self-criticism. This was accompanied by a reduction in parasympathetic activation, indicated by decreased HRV. In addition, results indicate that this condition was associated with increased physiological arousal indexed by increases in HR and several-minutes-delayed reductions in skin-conduction level (inferring increased sympathetic activation). Increased HR and SCL and reduced HRV during this condition were significantly correlated with reduced self-reported self-compassion and positive affiliative affect and increased self-criticism. However, there was no evidence for significant mediation effects of these physiological responses for any of the self-report changes (see Supplemental Material). Our findings are in line with research that associates self-critical rumination with enhanced cardiovascular and sympathetic activation (Dickerson & Kemeny, 2004; Mandell et al., 2014; Siegle & Thayer, 2003) and reduced HRV (Woody, McGeary, & Gibb, 2014).

The exercise designed to stimulate the drive system led to a psychophysiological response pattern different from the two self-compassion conditions. Increases in positive and a reduction in negative affective states were accompanied by higher self-reports of feeling energized and a gradual increase in HR and decrease in HRV in the last 2 min of the exercise. This is not surprising, given that the drive system of the tripartite model has been described as being one of behavioral activation (Depue & Morrone-Strupinsky, 2005; Gilbert, 2009, 2014). It is characterized by a high incentive salience of a stimulus that motivates the individual to act in order to achieve goals, and is also described as the “wanting” component of the reward system (Berridge & Robinson, 2016; Gray, 1987; Panksepp, 1998). Importantly, this dimension of positive affect has also been linked to social behaviors of comparison, competitiveness, or status seeking (Depue & Morrone-Strupinsky, 2005; Gilbert, 2009, 2014). There is increasing acknowledgment that the brain systems underpinning this type of positive affect may be associated with problematic mental states, including mania (Alloy & Abramson, 2010) and addiction (Berridge & Robinson, 2016), and health problems, such as chronic stress (Pani, 2000).

Similar to the self-compassion inductions, the positive excitement exercise led to increased self-reported levels of state self-compassion, positive affiliative affect, and decreased state levels of self-criticism. In contrast, the positive excitement condition was the only condition that induced higher levels of feelings energized. Previous research had psychometrically distinguished between excited and calm positive affect systems (Gilbert et al., 2009; Kelly et al., 2012), and our findings are in partial agreement with this. In line with Wood et al. (2003), we had expected that it would increase positive affect but that significant changes in state self-compassion and positive affiliative affect would be observed only in the self-compassion conditions. Effects are indeed larger and postinduction group differences are in support of this assumption (see Supplemental Materials). It should be noted that we did not use the widely utilized positive and negative affect schedule (Crawford & Henry, 2004), which assesses excited positive affect in more detail, but we used a small number of visual analogue scales with a main focus on studying calm and affiliative positive affect in order to reduce testing time for repeated state assessments in an already-long paradigm developed for patient studies.

Finally, the neutral control condition (supermarket scenario) did not significantly affect the self-report and physiological measures. Although there is existing support for it (Kelly et al., 2012), to our knowledge this is the first study that fully tested and confirmed Gilbert’s (2009) tripartite model complementing self-report with psychophysiological measures. Future research should replicate our findings and may potentially identify additional behavioral or physiological indicators. For example, the inclusion of additional cardiovascular parameters could help further tease apart the complex interplay between sympathetic and parasympathetic activation (Berntson, Cacioppo, & Quigley, 1991). In addition, it would be interesting to see if there are differential activation patterns in brain areas associated with compassion, reward, and self-referential processing when individuals follow our self-compassion and positive excitement exercises. Klimecki, Leiberg, Ricard, and Singer (2014) have studied psychological and fMRI correlates of empathy and compassion training and identified different brain circuitries and self-reported affective states for both.

### Theoretical implications for the construct of self-compassion

Our findings have several theoretical implications. As discussed above, our findings are in line with previous research that suggests that self-compassion could be beneficial for mental health and well-being because it activates an emotion-regulation system that has been associated with calm and content positive affect, soothing, and social affiliation. We also suggested that facilitating self-compassion may act via addressing cognitive vulnerability by reducing negative self-bias and increasing positive self. Our findings thus give partial support to the current theoretical understanding of self-compassion (Neff, 2003b) and compassion (Engen & Singer, 2015; Gilbert, 2009), but also social engagement (Porges, 2007), social connectedness (Cacioppo et al., 2000), and secure attachment (Mikulincer & Shaver, 2007a, 2007b).

It has to be noted that we found the pattern of low-arousal parasympathetic positive affect in a healthy sample in a nonthreatening context. We therefore concluded that activating a self-compassionate state appears to resemble the physiological pattern of relaxation or related techniques (e.g., Kanji et al., 2006; Yuksel et al., 2013; Zeidan et al., 2010). It remains to be studied if activating or retaining a compassionate, kind attitude toward the self while being in distress or during stress recovery would be accompanied by a similar pattern. This is one important feature of self-compassion that we did not test in our study, because without having established the exercises’ effectiveness to induce calm, content, affiliative state in a nonthreatening context first, we would not be able to understand potentially nonsignificant or opposite findings during a stress condition. In this vein, our results of reduced HR are in contrast to Lutz, Greischar, Perlman, and Davidson (2009), who reported increased HR during compassion meditation in experienced meditators, as compared to novices. Whereas participants in Lutz et al. (2009) were invited to cultivate compassion toward images of individuals who displayed suffering, in our self-compassion condition, no negatively-valenced material was presented. Counterintuitive higher HR in experienced meditators as compared to novices should be considered in context of their greater brain activation in areas implicated in empathy and compassion as an indicator of effective emotion regulation. In particular, advanced meditators will not suppress, but accept negative emotions and disengage from their own distress to direct compassion toward another individual in the presence of suffering. This latter aspect has been described as one important component of self-compassion; for example, the ability to exert self-kindness in times of adversity (Strauss et al., 2016). We would hypothesize that the experienced meditators in the Lutz et al. (2009) study may show higher HRV, a parameter (discussed in detail, above) that has been associated with more adaptive emotion regulation in the presence of psychosocial stressors.

Next, research should investigate psychophysiological response patterns when self-compassion is cultivated in stressful situations when the self is threatened. Interestingly, Gilbert et al. (2008) found that relaxed and secure positive affect are significantly correlated but form distinct subscales in a newly developed self-report measure which could be relevant for contexts in which compassion is directed to the self in psychosocial threat conditions. Extending Lutz et al.’s (2009) research, exploring additional physiological measures such as skin conductance to identify possible emotion suppression (Roisman, Tsai, & Chiang, 2004), and HRV as an indicator of effective moment-to-moment physiological self-regulation (Hildebrandt et al., 2016; Woody et al., 2014), is warranted.

### Limitations and avenues for future research

This study has several limitations. For instance, the age range of participants was very narrow. In addition, in general, the sample was very homogenous in terms of the trait levels of self-compassion and self-criticism. Future studies should be conducted to investigate whether the findings extend across more diverse samples. Another limitation is the lack of respiratory data, as it has been demonstrated that breathing might affect cardiac vagal tone (Ritz & Dahme, 2006). Hence, HRV changes could be attributable to changes in breathing rate or depth. However, physical demands were kept constant throughout the study, and care was taken that none of the experimental manipulations focused on the breath, making the influence of breathing on the HRV results unlikely. Moreover, there is recent evidence that respiration can be neglected when investigating the association between HRV and inhibition (Park, Van Bavel, Vasey, & Thayer, 2013). In addition, our LKM-S did not allow us to distinguish directly between the self-reported effects of self-focus and other-focused compassion, whereas sustained changes in the psychophysiology responses throughout the LKM-S suggest similar response patterns to both self-focused and other-focused compassion. However, our study design did not allow us to investigate the specific interplay between those two forms of compassion because we did not counterbalance the order of other- versus self-directed compassion.

Although the sample size in this study was based on a priori power calculation for medium effect sizes in mixed measures ANOVAs and the recruitment target was met, a larger sample size may have been desirable. Overall, a sample of 135 is considered to be a good sample size for growth curve modeling (Curran, Obeidat, & Losardo, 2010) or mediation analyses for medium-to-large effects (Fritz & MacKinnon, 2007). However, some of the effects were small-to-medium rather than medium and failed to reach significance, and thus a replication in a larger sample is warranted to check the robustness of our effects.

Finally, the findings raise theoretical questions that may open avenues for future research. First, the results of this study suggest that both the direct and indirect self-compassion inductions induced similar psychophysiological response patterns. However, this does not allow the conclusion that they are working via similar processes because there may be individual differences in people’s ability to engage with more direct and indirect approaches to cultivate self-compassion. Second, given the small-to-medium effect sizes in this study, the mechanism we focused on may not be the only one by which self-compassion exerts its positive effect. For example, there is an emerging consensus that automatic and elaborate self-referential processing biases toward negative information, and their neural underpinnings play an important role for the maintenance of mental health problems such as depression (e.g., Shestyuk & Deldin, 2010). It is less-well understood if the facilitation of self-compassion also reduces negative self-referential processing, as is often reported in individuals with depression. Future research should therefore investigate the potential role of self-compassion in self-referential processing.

## Conclusions

The current study extends previous research on the effects of cultivating self-compassion by employing an experimental approach that combined self-report state measures, psychophysiology, and advanced statistical methods to study the time course of responses to two newly developed self-compassion conditions in comparison to three control conditions mapped on the tripartite model of affect regulation. It thus keeps with recent calls for self-compassion studies that do not rely on self-report alone (e.g., Hofmann et al., 2011). We conclude that self-compassion reduces negative self-bias and activates a content and calm state of mind with a disposition for kindness, care, social connectedness, and the ability to self-soothe when stressed. Our paradigm might serve as a basis for future research in analogue and patient studies addressing several important outstanding questions.

## Supplemental Material

KirschnerFigS1 – Supplemental material for Soothing Your Heart and Feeling Connected: A New Experimental Paradigm to Study the Benefits of Self-Compassion Click here for additional data file. Supplemental material, KirschnerFigS1 for Soothing Your Heart and Feeling Connected: A New Experimental Paradigm to Study the Benefits of Self-Compassion by Hans Kirschner, Willem Kuyken, Kim Wright, Henrietta Roberts, Claire Brejcha and Anke Karl in Clinical Psychological Science

KirschnerFigS2 – Supplemental material for Soothing Your Heart and Feeling Connected: A New Experimental Paradigm to Study the Benefits of Self-Compassion Click here for additional data file. Supplemental material, KirschnerFigS2 for Soothing Your Heart and Feeling Connected: A New Experimental Paradigm to Study the Benefits of Self-Compassion by Hans Kirschner, Willem Kuyken, Kim Wright, Henrietta Roberts, Claire Brejcha and Anke Karl in Clinical Psychological Science

KirschnerFigS3 – Supplemental material for Soothing Your Heart and Feeling Connected: A New Experimental Paradigm to Study the Benefits of Self-Compassion Click here for additional data file. Supplemental material, KirschnerFigS3 for Soothing Your Heart and Feeling Connected: A New Experimental Paradigm to Study the Benefits of Self-Compassion by Hans Kirschner, Willem Kuyken, Kim Wright, Henrietta Roberts, Claire Brejcha and Anke Karl in Clinical Psychological Science

KirschnerFigS4 – Supplemental material for Soothing Your Heart and Feeling Connected: A New Experimental Paradigm to Study the Benefits of Self-Compassion Click here for additional data file. Supplemental material, KirschnerFigS4 for Soothing Your Heart and Feeling Connected: A New Experimental Paradigm to Study the Benefits of Self-Compassion by Hans Kirschner, Willem Kuyken, Kim Wright, Henrietta Roberts, Claire Brejcha and Anke Karl in Clinical Psychological Science

Kirschner_Supplemental_Material – Supplemental material for Soothing Your Heart and Feeling Connected: A New Experimental Paradigm to Study the Benefits of Self-Compassion Click here for additional data file. Supplemental material, Kirschner_Supplemental_Material for Soothing Your Heart and Feeling Connected: A New Experimental Paradigm to Study the Benefits of Self-Compassion by Hans Kirschner, Willem Kuyken, Kim Wright, Henrietta Roberts, Claire Brejcha and Anke Karl in Clinical Psychological Science

## Notes

1. In a body scan practitioners are guided to direct attention to their body sensations.

2. During a breath-awareness practice, attention is brought to and sustained on the sensations of the breath.
