# Supplementary figures and images for: Soothing Your Heart and Feeling Connected: A New Experimental Paradigm to Study the Benefits of Self-Compassion
Source: Clin Psychol Sci. 2019 Feb 6;7(3):545–65. doi: 10.1177/2167702618812438 (PMC7324152; doi:10.1177/2167702618812438)

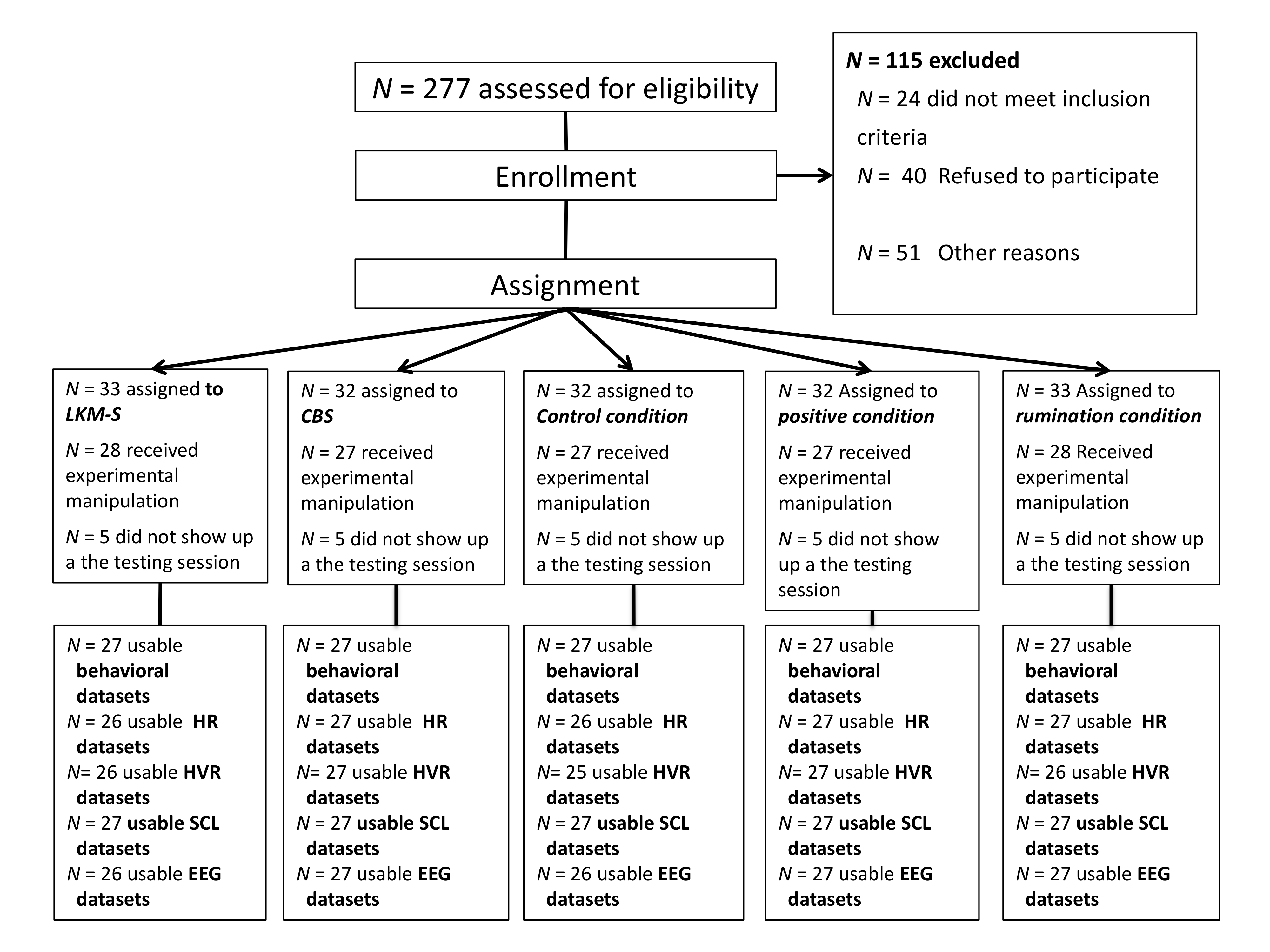

Supplement: KirschnerFigS1 – Supplemental material for Soothing Your Heart and Feeling Connected: A New Experimental Paradigm to Study the Benefits of Self-Compassion [file KirschnerFigS1.jpg]

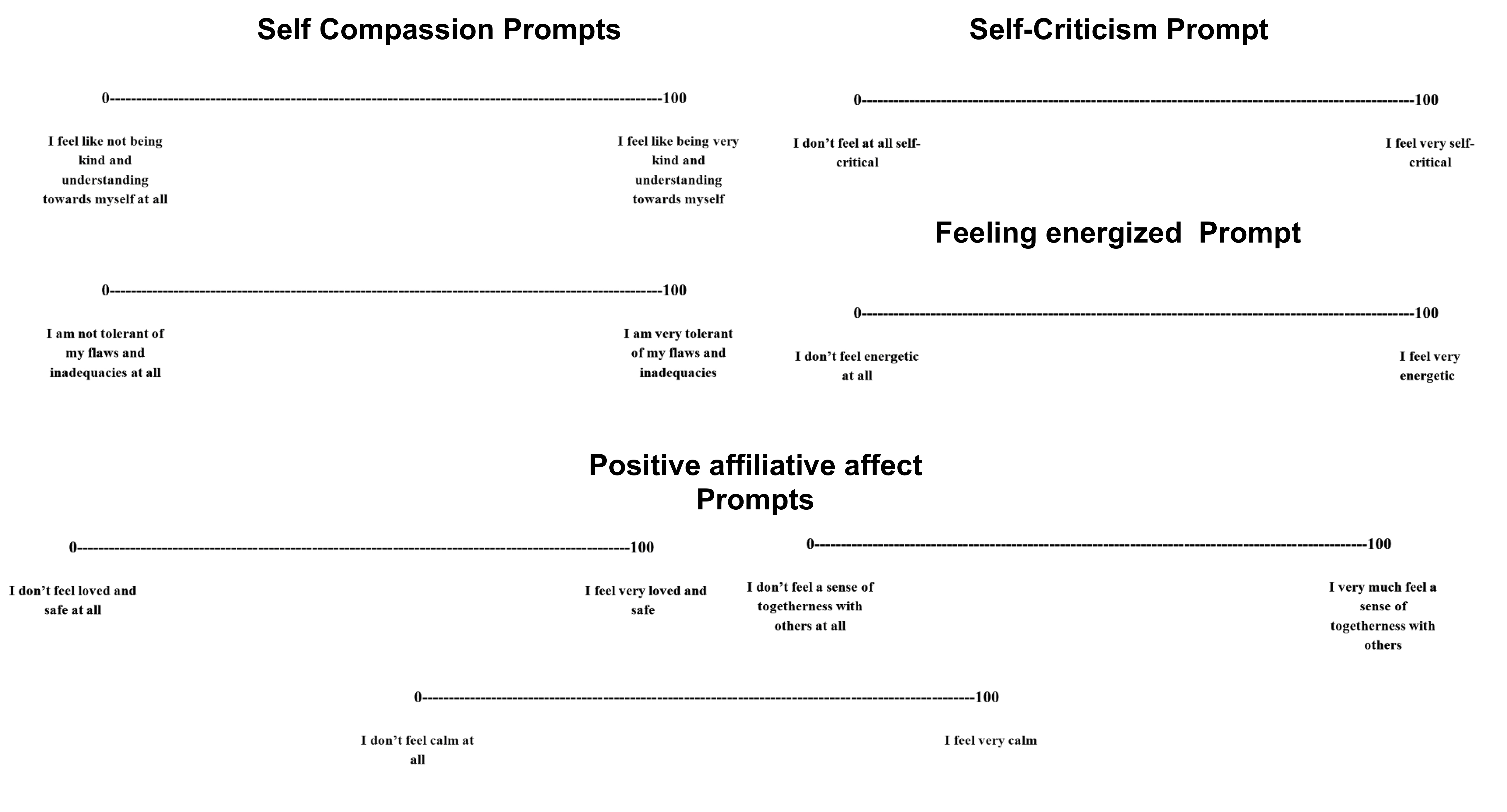

Supplement: KirschnerFigS2 – Supplemental material for Soothing Your Heart and Feeling Connected: A New Experimental Paradigm to Study the Benefits of Self-Compassion [file KirschnerFigS2.jpg]

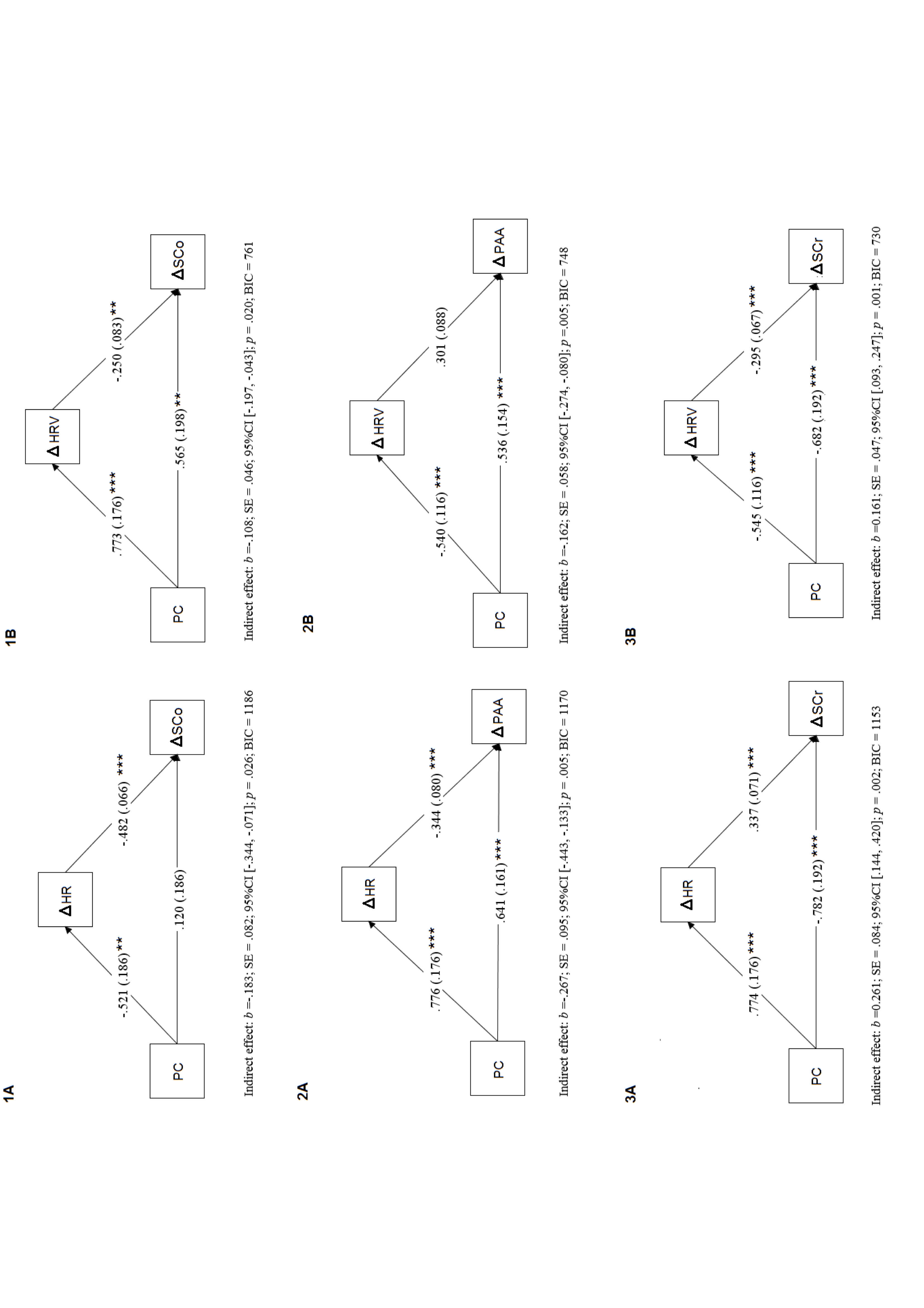

Supplement: KirschnerFigS3 – Supplemental material for Soothing Your Heart and Feeling Connected: A New Experimental Paradigm to Study the Benefits of Self-Compassion [file KirschnerFigS3.jpg]

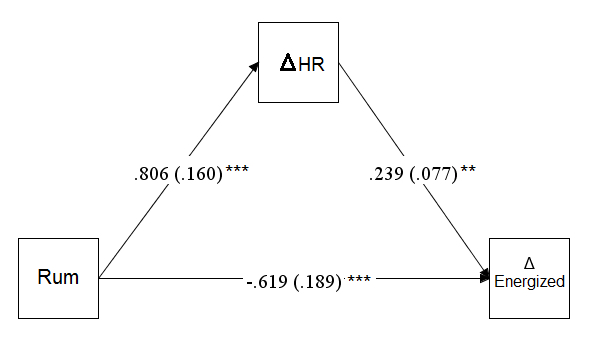

Supplement: KirschnerFigS4 – Supplemental material for Soothing Your Heart and Feeling Connected: A New Experimental Paradigm to Study the Benefits of Self-Compassion [file KirschnerFigS4.jpg]
