# Supplementary material for: Soothing Your Heart and Feeling Connected: A New Experimental Paradigm to Study the Benefits of Self-Compassion
Source: Clin Psychol Sci. 2019 Feb 6;7(3):545–65. doi: 10.1177/2167702618812438 (PMC7324152; doi:10.1177/2167702618812438)
Supplement: Kirschner_Supplemental_Material – Supplemental material for Soothing Your Heart and Feeling Connected: A New Experimental Paradigm to Study the Benefits of Self-Compassion [file Kirschner_Supplemental_Material.pdf]

Supplemental Material

Participant flow diagram

Figure S1 depicts the flow of participants through the study.

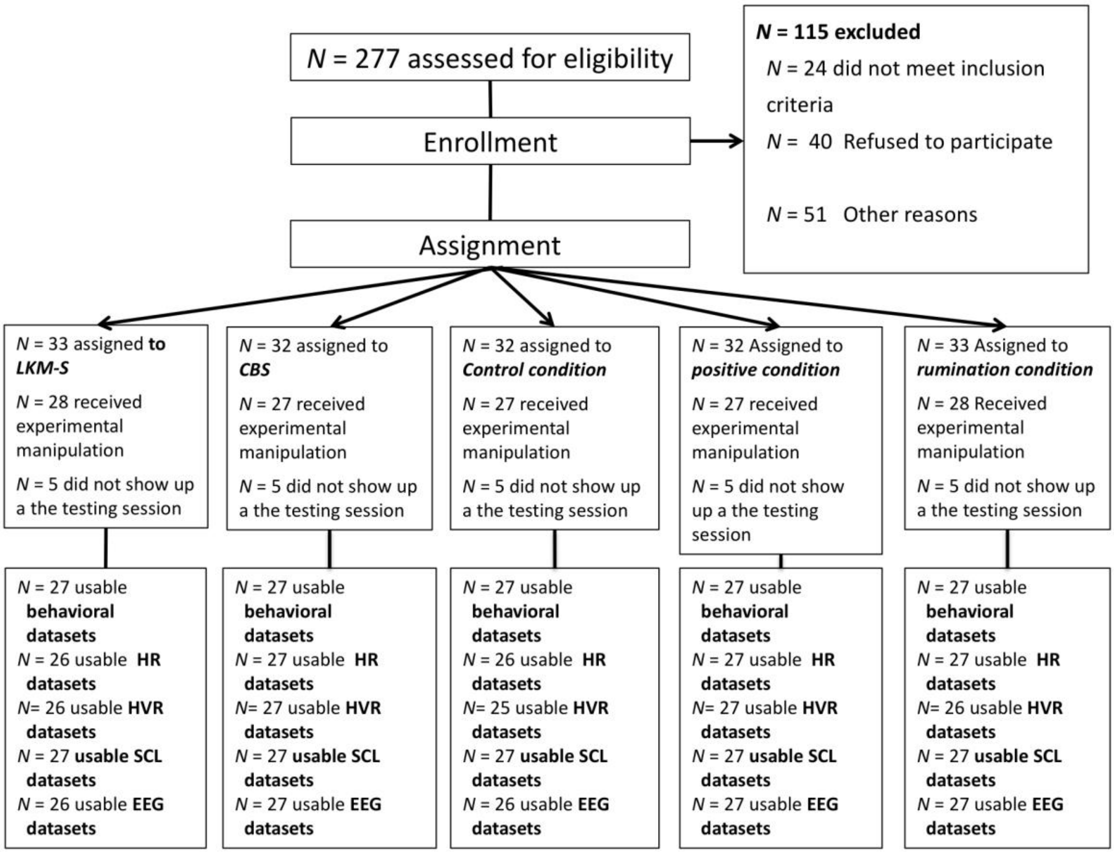

Figure S1. Participant flow diagram. *Note:* Reasons for exclusion of physiological dataset were poor data quality. In addition one participant in the LKM and one in the rumination condition could not follow the instructions of the audio-exercises. They have been excluded from subsequent analyses. In addition, some physiological data was lost for the post induction exercise due to technical difficulties (LKM: 5 data sets (18.5 %); Body Scan: 1 data set (3.7 %); Control Condition: 6 data sets (22.2 %); positive Condition: 8 data sets (29.63 %); Rumination: 2 datasets (7.4 %)). HR, Heart Rate; HVR, Heart Rate Variability; SCL, Skin Conductance Level.

Visual prompts for the visual analogue scales used in the manipulation checks

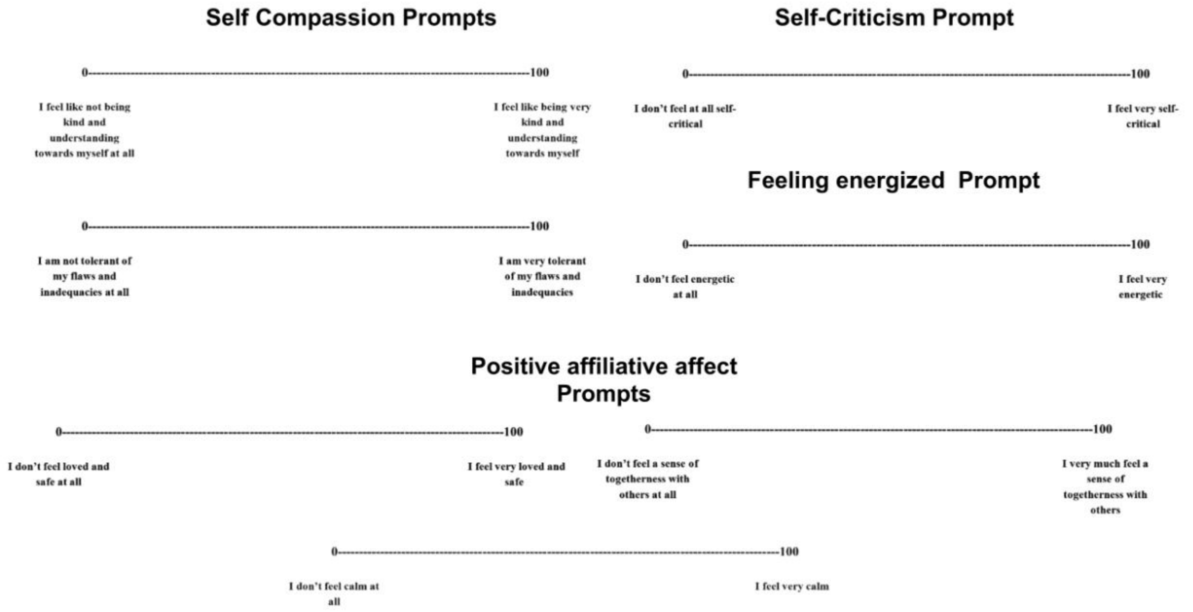

Figure S2. Visual prompts for the visual analogue scales used in the manipulation checks.

ANCOVA for VAS/ Self-report measures

Self-Compassion. An ANCOVA on post-induction scores, using pre-induction scores as the covariate was significant, $F(4, 129) = 16.40$, $p < .001$, $\eta^2_p = .34$. Sidak corrected pairwise comparisons revealed that post-induction - compared to the neutral condition – participants assigned to the body scan reported higher state self-compassion scores ($p = .001$), a similar pattern could be found for the LKM ($p = .025$), whereas the rumination induction was associated with lower state self-compassion scores ($p = .037$). Moreover, there was a trend for higher state self-compassion scores for the positive excitement condition as compared to the neutral condition ($p = .058$).

Self-criticism. Similar to the state self-compassion findings, there was a group effect on post-induction self-criticism scores after controlling for pre-induction self-criticism scores, $F(4, 129) = 16.17$, $p < .001$, $\eta^2_p = .33$. The self-criticism ratings are depicted in Figure 1B.

The Sidak-corrected pairwise comparisons revealed that only the rumination condition lead to increased state levels of self-criticism as compared to the neutral condition ($p > .001$), Moreover there was a trend towards lower state levels of self-criticism after the body scan when compared with the neutral condition ($p = .073$). No difference between the neutral condition, t he LKM, and the positive excitement condition emerged (all $p > .05$).

Positive affiliative affect. An ANCOVA on post-induction scores, using pre-induction scores as the covariate was significant, $F(4, 129) = 26.50$ $p < .001$, $\eta^2_p = .45$. Pairwise comparisons revealed that compared to the neutral condition participants assigned to the LKM demonstrated higher levels of state positive affiliative affect ($p = .001$). In contrast, being in the rumination condition was associated with lower levels of positive affiliative affect as compared to participants assigned to the neutral condition ($p > .001$). No other significant post-induction group differences emerged ($p > .05$).

Feeling energized. An ANCOVA on post-induction scores, using pre-induction scores as the covariate was significant, $F(4, 129) = 7.58$ $p < .001$, $\eta^2_p = .19$. Sidak corrected pairwise comparisons revealed that compared to the neutral condition only participants assigned to positive excitement condition demonstrated higher energy levels ($p = .001$). No other group differences yielded significance (all $p > .05$).

Pre – Post baseline changes in physiology

A follow-up ANCOVA on physiological post-audio-exercise-baseline measures, using pre-audio-exercise-baseline-scores as the covariate did not yield a significant group difference for skin conductance level ($F(4,107) = 0.44$, $p = .777$, $\eta^2_p = .01$), heart rate ($F(4,109) = 0.72$, $p = .578$, $\eta^2_p = .03$), and heart rate variability (F(4,101) = 1.54, $p = .195$, $\eta^2_p = .06$). Physiological post-audio-exercise-baseline-measures are depicted in Table 1.

Zero order correlations

In order to determine statistical significance between correlation coefficients, the Z-test for dependent correlations within a population (Meng, Rosenthal, & Rubin, 1992; Steiger, 1980) was used applying the FZT calculator (Garbin, http://psych.unl.edu/psycrs/statpage/comp.html). A Steiger's Z ≥ 1.96 indicates that correlation coefficients differ significantly at the p <.05 level.

Both self-compassion conditions were significantly correlated with HR reduction and HRV increase, as well as with self-reported increase in self-compassion and positive affiliative affect (absolute r coefficients ranging from .20 to .39). In addition, the LKM-S but not CBS was also associated significantly with SCL reduction (r = -.18) whereas the CBS but not LKM-S was associated with significantly reduced self-criticism (r = -.27) and feeling energized (r = -.17). In both instances, the correlation coefficients were not significantly different though (Steiger's Z = 1.54; p = .13 for SCL and Z = 1.60; p = .11 for change in self-criticism).

In contrast, the opposite pattern of significant correlations was found for the rumination condition (absolute r coefficients ranging from .31 to .61), with the exception of a significant association of reduced feeling energized ratings (r = -.17) similar to the CBS. All correlation coefficients were significantly different from those for LKM-S with self-report or physiological response and for CBS with self-report or physiological response (Steiger's Z ranging from Z = 4.26, p < .001 to Z = 7.57, p < .001).

Being in the positive condition was significantly correlated with change in self-reported self-criticism (r = -.21) and feeling energized (r = .41) but not change in self-compassion or positive affiliative affect with significantly different correlation coefficients between being in positive condition and self-compassion change (Steiger's Z = 2.38; p = .017) and positive affiliative affect change (Steiger's Z = 2.40; p = .018). More interestingly,

it was significantly associated with similar physiological response changes as in the rumination condition; i.e., increased HR and reduced HRV (absolute *r* coefficients ranging from .22 to .31) and thus significantly different from correlation coefficients between HR and HRV changes and being in the LKM-S and CBS conditions (Steiger's Z ranging from Z = 3.58, $p < .001$ to Z = 5.16, $p < .001$). Comparison of correlations coefficients for change in self-report with LKM-S and CBS revealed smaller effects. The comparison between correlation coefficients for change in positive affiliative affect and LKM-S with that for change in positive affiliative affect and positive condition approached significance (Steiger's Z = 1.90; $p = .054$). Moreover, the correlation coefficient for change in feeling energized and the positive condition was significantly different to the correlation coefficient for change in feeling energized and the rumination condition (Z = 2.40, $p = .014$) and the CBS (Z = 2.37, $p = .016$).

Heart rate variability change as mediator for effects of self-compassion inductions on self-report

The only significant mediation effect of HRV was found for the effect of LKM on reduction of self-criticism: a significant indirect effect was observed, $b = -.225$; SE = .100, 95%CI [-.427 - -.095], $p = .024$; BIC = 725, but the direct effect of LKM on change in self-criticism was no longer significant, $b = -.065$; SE = .164, 95%CI [-.326 - .222], $p = .693$; BIC = 725. The direct effect of HRV increase of self-criticism reduction was significant, $b = -.227$; SE = .100, 95%CI [-.341 - -.070], $p = .002$; BIC = 725, as was the effect of LKM on HRV increase, $b = .991$; SE = .245, 95%CI [.564 - 1.368], $p < .001$; BIC = 725. This suggests that the effect of being in the LKM condition on reduction in self-criticism is fully mediated by increase in HRV.

Heart rate change as mediator for effects of positive control induction on self-report

Change in state self-compassion. The direct path was not significant but a significant indirect effect was identified (see Figure S3, panel 1A), suggesting a full mediation, i.e.; that the positive condition exerted its effect on increasing self-compassion only via a reduction in HR.

Change in state positive affiliative affect. There were significant direct and indirect effects (see Figure S3, panel 2A) suggesting that a partial mediation, i.e.; the positive condition exerted its effect on increase in positive affiliative affect direct and via a smaller increase in HR.

Change in state self-criticism. There were again significant direct and indirect effects (see Figure S3, panel 3A) suggesting a partial mediation; i.e., that the positive condition exert its effect on decreasing self-criticism direct and via a reduction in HR.

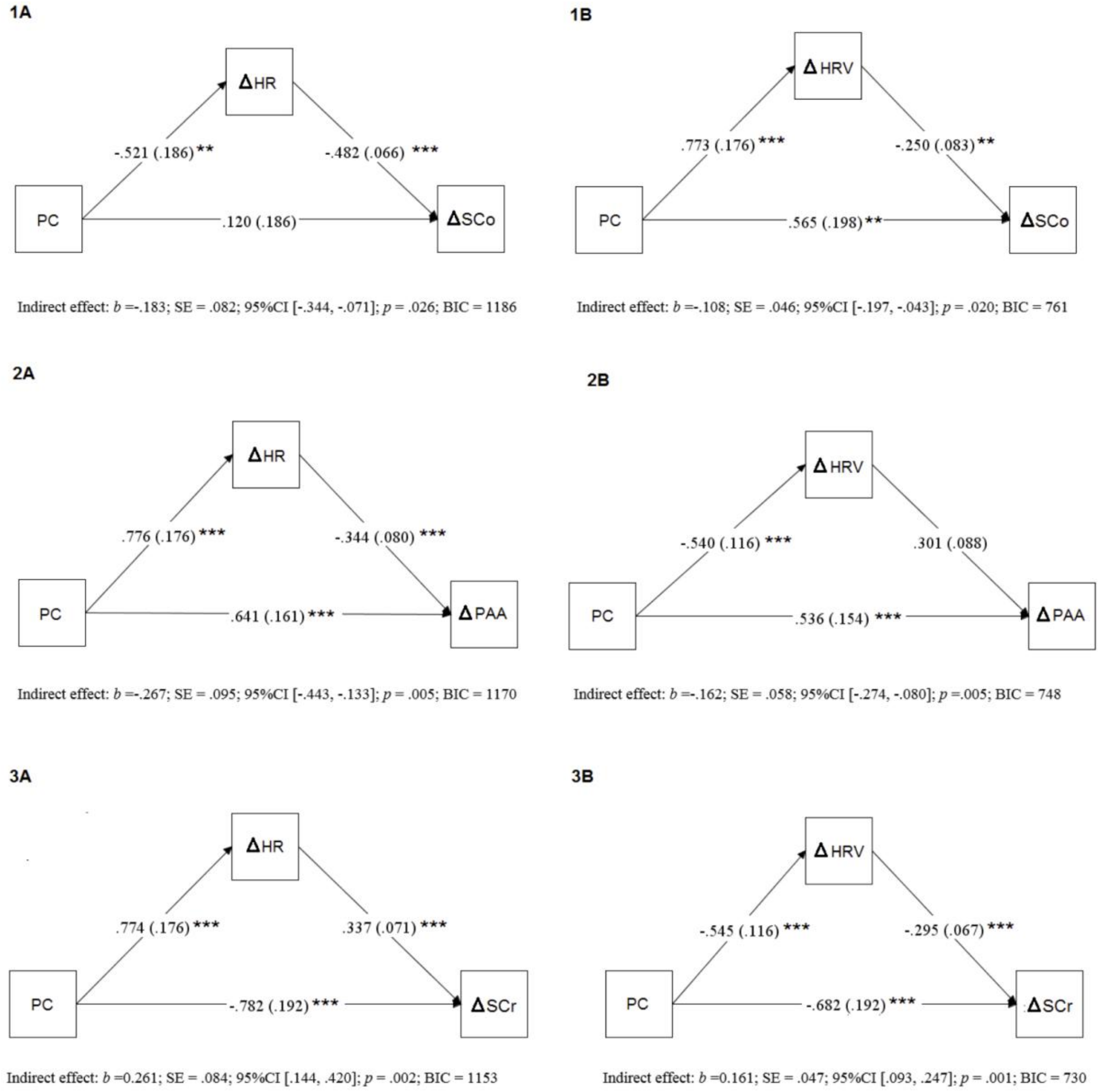

Figure S3. Mediation analyses for HR and HRV responses as mediators for the effect of the positive control condition on changes in self-report; HR = heart rate; HRV = heart rate variability; PC = positive control condition; SCo = self-compassion; PAA = positive affiliative affect; SCr = self-criticism.

Heart rate variability change as mediator for effects of positive control induction on self-report

Change in state self-compassion. There were significant direct and indirect effects (see Figure S3, panel 1B) suggesting a partial mediation, i.e.; the positive condition exerted its effect on increase in self-compassion direct and via an increase in HRV.

Change in state positive affiliative affect. There were significant direct and indirect effects (see Figure S3, panel 2B) suggesting a partial mediation, i.e.; the positive condition exerted its effect on increase in positive affiliative affect direct and via an increase in HRV.

Change in state self-criticism. There were again significant direct and indirect effects (see Figure S3, panel 3B) suggesting a partial mediation; i.e., that the positive condition exerted its effect on decreasing self-criticism direct and via an increase in HRV.

Mediation analyses for rumination condition

There were only significant direct and indirect effects for the effect of being in the rumination condition to feeling energised (Figure S4), suggesting a partial mediation; i.e., that self-critical rumination exerts its effect on decreased feeling energised directly and indirectly via an increase in HR (b = 0.19, SE = 0.08, p = .010). No other indirect effects reached significance.

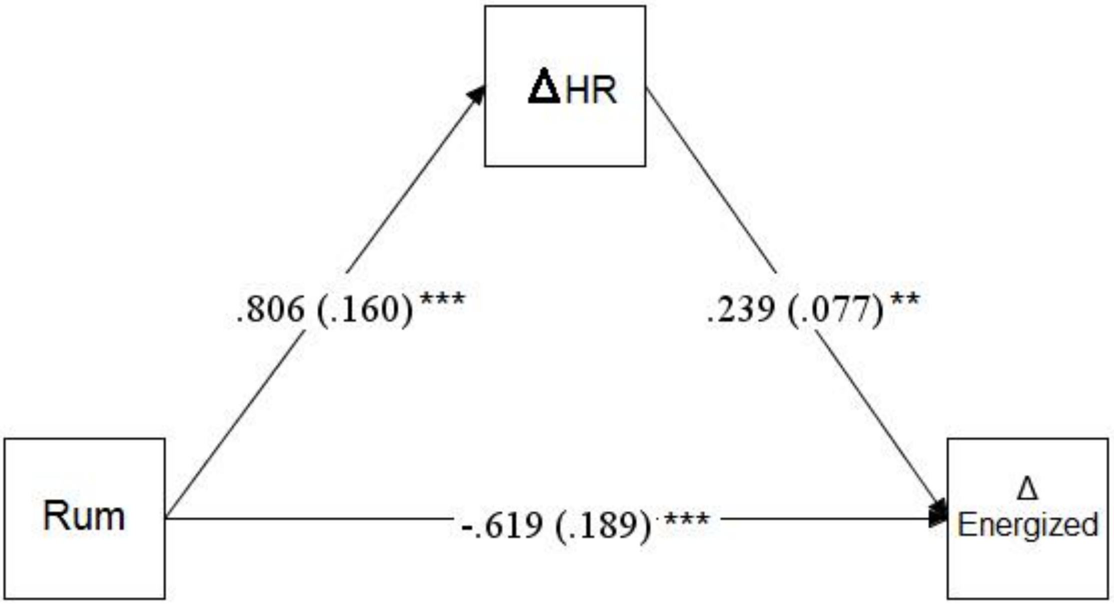

Figure S4. Mediation analyses for HR responses as mediators for the effect of the rumination condition on changes in self-report; HR = heart rate; RUM = Rumination Condition; Energized = residualized gain score for feeling energized ratings

References

Meng, X.-l., Rosenthal, R., & Rubin, D. B. (1992). Comparing correlated correlation coefficients. *Psychological Bulletin, 111*(1), 172-175. doi:10.1037/0033-2909.111.1.172

Steiger, J. H. (1980). Tests for comparing elements of a correlation matrix. *Psychological Bulletin, 87*(2), 245-251. doi:10.1037/0033-2909.87.2.245
